# A pedigree-based genetic appraisal of Boxer ARVC and the role of the Striatin mutation

**DOI:** 10.1136/vr.102821

**Published:** 2015-02-06

**Authors:** B. M. Cattanach, J. Dukes-McEwan, P. R. Wotton, H. M. Stephenson, R. M. Hamilton

**Affiliations:** 1 MRC Mammalian Genetics Unit, Harwell Science and Innovation Campus, Oxfordshire OX11 0RD, UK; 2 University of Liverpool Small Animal Teaching Hospital, Leahurst, Chester High Road, Neston CH64 7TE, UK; 3 University of Glasgow School of Veterinary Medicine, Bearsden Road, Glasgow G61 1QH, UK; 4 HS Cardiology Ltd, Barnfield Farm, Tunstall, Carnforth LA6 2RU, UK; 5 The Hospital for Sick Children, 555 University Avenue, Toronto, Ontario M5G 1X8, Canada

**Keywords:** Cardiomyopathy, Genetics, Dogs

## Abstract

The objective of this paper was to investigate by pedigree-based genetic means the origins and inheritance of arrhythmogenic right ventricular cardiomyopathy (ARVC) in UK Boxers and assess the role of the proposed causal mutation in the gene, Striatin (STRN). All ARVC cases traced back to a small number of imported American dogs deriving from the group of Boxers studied by Harpster (1983) to define the disease, strongly suggesting that the disease is the same in the two countries. Dogs with and without the STRN mutation were found in both ARVC affected and normal Boxers showing that the mutation is not responsible for the disease. Evidence was found that the STRN mutation is, however, genetically linked with the gene responsible on the same chromosome. The linkage implies that the two genes can separate by meiotic recombination such that both ARVC-affected and ARVC-unaffected lines of dogs may carry either the STRN mutation or its wild-type allele. These have been found. Homozygotes for the STRN mutation tended to be severely affected at early ages, suggesting that there is an interaction between the known effects of the STRN mutation on the cardiomyocyte and ARVC.

## Introduction

Cardiomyopathy in Boxers is a late-onset familial disease that has been known in America for at least 30 years. It was first studied in detail by Harpster (1983, 1991) who described three clinical categories: Cat. 1 showing no clinical signs but having ventricular arrhythmias, Cat. 2 showing arrhythmias associated with clinical signs (e.g. syncope), and the most severe, Cat. 3, showing evidence of congestive heart failure, often biventricular, with coughing, lethargy and ascites, with or without arrhythmias or syncopal episodes. Cat. 3 shows phenotypic similarities to canine idiopathic dilated cardiomyopathy (DCM; Harpster 1991). It has not been clearly established whether the severity categories represent a progression of the disease, or if they have different aetiologies. The Boxer cardiomyopathy has subsequently been well characterised and proposed as a naturally occurring animal model of the important similar human condition, arrhythmogenic right ventricular cardiomyopathy (ARVC), and given this name (Basso and others 2004).

Pedigree analysis has suggested an autosomal dominant mode of inheritance with incomplete penetrance (Meurs and others 1999), consistent with human ARVC, but the published data are limited. A number of different genetic mutations appear to cause the disease in humans (Iyer and Chin 2013), and the finding in ARVC-affected humans of mutations in genes coding for desmosomal proteins has led to the screening and exclusion of most of these in Boxers (Meurs and others 2007). However, one genome-wide association study (GWAS) with subsequent gene sequencing identified a deletion mutation in an untranslated region of the Striatin gene (STRN), located on the *canis familiaris* chromosome (CFA) 17, which was associated with Boxer ARVC. This gene was a compelling candidate as the protein had been shown to localise to the intercalated disc and co-label with certain desmosomal proteins (Meurs and others 2010). However, 4 of 61 cases investigated by Meurs and others (2010) did not have the STRN mutation and it was present in 9 of 38 of their controls, the latter attributed to the incomplete penetrance. Subsequent reports have indicated further exceptions (Meurs and others 2014, Oxford and others 2014) and anomalous results have been obtained in veterinary practices with the commercially available ARVC test (e.g. www.laboklin.co.uk/laboklin/GeneticDiseases.jsp), which is based on the STRN mutation being the cause of the disease (Dukes-McEwan and others 2010). The STRN mutation has recently been shown to be associated with the Cat. 3 phenotype (Meurs and others 2013). The existence of ARVC cases without the STRN genotype may indicate genetic heterogeneity, or phenocopies due to non-genetic causes, or a different genetic cause of Boxer ARVC.

Boxer ARVC was first clearly recognised in the UK in the late 1990s (Wotton 1999) and it has been assumed, but not proven, that the disease is the same as that in the USA. The disease categories (phenotypes) have commonly been found in UK Boxers (Palermo and others 2011), some without the STRN mutation (Dukes-McEwan and others 2010).

This study does not use experimental protocols, but seeks to interpret the available breeding and veterinary evidence obtained from Boxer breeders and practising veterinary surgeons throughout the UK, with inclusion of like material collected at the Liverpool and Glasgow veterinary schools. We present pedigree analyses to (1) show that UK and American Boxer ARVC are the same, (2) verify that the mode of inheritance is that of an autosomal dominant with incomplete penetrance, (3) most importantly, we show that the STRN mutation is not the cause of the disease phenotype but is linked to the responsible gene on the same chromosome, and (4) illustrate that the three categories of Boxer ARVC have the same genetic basis but with an STRN effect possibly interacting to increase severity.

## Material and methods

The material for this study comprised UK Boxers with ARVC together with selected normal dogs collected from the show section of the breed over the years 2000–2010. Extended pedigree analyses were conducted by BMC using Kennel Club records. Blood samples were taken for STRN testing and for other routine diagnostic testing and surplus blood was stored in EDTA for genomic DNA extraction and used in an ongoing GWAS study (R. M. Hamilton, personal communication).

The dogs investigated derived from two sources. ARVC diagnoses in one group (BMC) were provided by practising veterinary surgeons’ or veterinary cardiologists’ reports, with diagnoses (made as below) verified by review of clinical records by PRW. The other group, referred directly to Glasgow (PRW) and Liverpool (JD-M and HMS) Veterinary Schools, were clinically diagnosed based on echocardiography, 24 h ambulatory electrocardiography (Holter) data (24AECG) according to the criteria specified by Meurs (2004), and exclusion of other causes of arrhythmia. They were then assigned to one of the three Harpster categories of severity (Harpster 1983, 1991).

For the pedigree studies, verification that cases from both sources specifically represented inherited disease was established by their presence in recognised family groups (BMC). Relationship was the key criterion for selection of all material for this genetic study. Rescue dogs and other ARVC cases without pedigrees were not included. Deduced normal Boxers were derived from sections of the breed defined by exclusion as free of the gene causing ARVC (or considered to be at low risk) through (1) the absence of any reported cases (see Results section) in a national veterinary screen for Boxer ARVC over the 10-year period of the study, (2) an absence of deduced ancestral sources of ARVC in their pedigrees (see Results) and (3) with support for this conclusion verified, as possible, by individual dogs in the study selected as each having several hundred progeny, none of which have been reported as ARVC cases, this again based on the national screen. For the veterinary school sources (JD-M, HMS and PRW), normal Boxers of ≥8 years old were also phenotyped (defined as phenotype-normal if unremarkable on echocardiography and 24AECG showed <50 VPCs/24 h), but for dogs from both sources (BMC and veterinary schools), classification of ARVC clear-by-pedigree remained the paramount criterion for normal (disease gene free or low risk). The mode of inheritance was ascertained by evaluation of pedigrees over several generations for evidence of inbreeding or the presence of ARVC-producing animals on both the sire's and dam's pedigrees. Either finding might suggest a recessive inheritance, and an absence in one or other could accord with a dominant. Sexes and severity of effect were noted to check for possible sex-linked inheritances.

Tracing the origins of the disease was complicated by the fact that the penetrance was extremely low such that the disease was rarely recognised in breeding animals. Thus, few parents were found to be affected even years after producing affected progeny. Pedigree analysis was therefore conducted by identifying such dogs through their production of affected progeny in litters with several different mates. Thereafter, lines of descent were distinguished by tracing these ARVC-producing dogs/bitches back through the pedigrees until they reached a common source. Such pedigree analyses were based on 183 ARVC-affected Boxers from the show section of the breed.

STRN genotyping of the initial Boxers was kindly conducted by Dr K. Meurs, using an amplified fragment length polymorphism, as previously described (Meurs and others 2013). Subsequent samples were genotyped by one of us (RMH) or at Antagene, courtesy of Dr Anne Thomas. Results were classified as being homozygous or heterozygous for the STRN mutation, or wild type (WT). Evidence on the association between the mutation and the disease phenotype was sought thereafter.

### Statistical methods

The age of diagnosis of ARVC in dogs and bitches was compared with an unpaired Student *t* test. Significance was accepted as P<0.05. The genotypes (homozygous and heterozygous for STRN, WT) were compared between groups with the χ^2^ test.

## Results

### General observations

Sexes were near-equally represented (54 per cent males, n=194). The range of expression as assessed by age of diagnosis was variable (<1 to over 10 years), this range even being found within a single litter. The median age of diagnosis was seven years. Age of diagnosis was greater in males than in females but not significantly so (P=0.082).

### Pedigree studies

**FIG 1:**
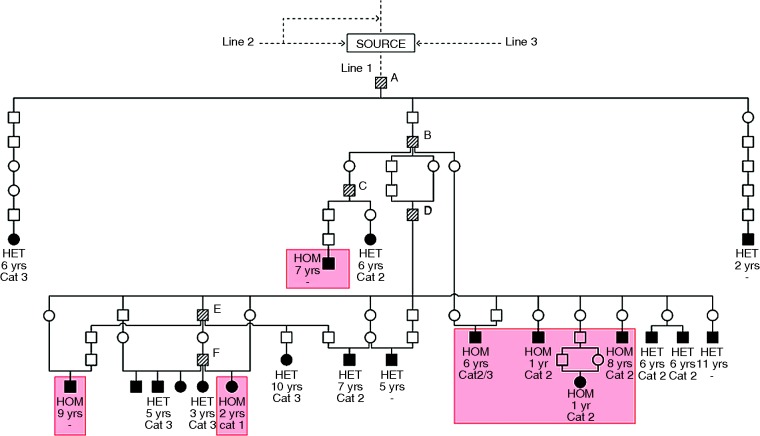
Pedigree chart showing line of descent of dogs transmitting arrhythmogenic right ventricular cardiomyopathy (ARVC) in line 1. Data are representative of the whole line 1 group (24 per cent of the total), and were selected primarily on the basis of having been STRN tested. Squares=males; circles=females; closed symbols=ARVC-affected dogs; shaded symbols=dogs that have produced several cases of ARVC; HOM=homozygous for the STRN mutation; HET=heterozygous for the STRN mutation; WT=dogs carrying the wild-type alleles of STRN. Key ARVC-producing dogs are marked A–F. Age of diagnosis is indicated and also ARVC severity category where attainable. Since all affected cases carry the STRN mutation, it is assumed that the source animal was of this genotype. Only the most informative dogs in terms of age of onset, severity category and STRN genotype are shown

The initial observation of ARVC in British Boxers was based on a cluster of closely inbred cases (Wotton 1999). As further cases were reported, lines of descent to a few individual ARVC-producing dogs could be seen (Fig 1, dogs B–F) and ultimately each of these could be shown to trace back to a single common ancestor (Fig 1, dog A), mostly through a grandson (Fig 1 dog B). Together, they are the source of all ARVC in this line of descent (line 1). Significantly, the older dog (A) was derived from Harpster's (1983) ARVC study source in America. Eighty-two dogs (47 males, 35 females) belonged in line 1.

**FIG 2:**
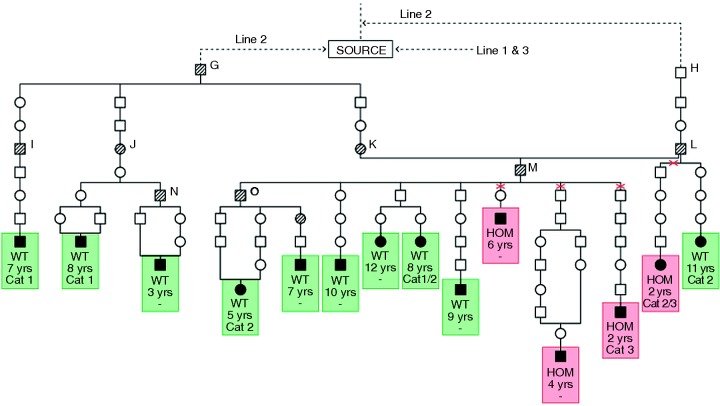
Pedigree chart showing line of descent of dogs transmitting arrhythmogenic right ventricular cardiomyopathy (ARVC) in line 2. Data are representative of the whole line 2 group (20 per cent of the total) and were selected primarily on the basis of having been STRN tested. Symbols are the same as in Fig 1. Key ARVC-transmitting dogs are marked G–O. Since the majority of dogs in this line are wild type (WT) for STRN, it is likely that the original source animals in this line were of this WT genotype. Possible sites of recombination are marked with red crosses. Only the most informative dogs in terms of age of onset, severity category and STRN genotype are shown

A second family line of dogs with ARVC was then recognised (line 2). This also traced back through a series of transmitting dogs (Fig 2, dogs G–O) to two North American imported males (Fig 2, dogs G and H), both again linking directly to the group studied by Harpster (1983). Seventy-six dogs (40 males, 36 females) belonged in line 2. Some ARVC cases of mixed line 1–line 2 origin were also found (5 males, 6 females; data not shown).

**FIG 3:**
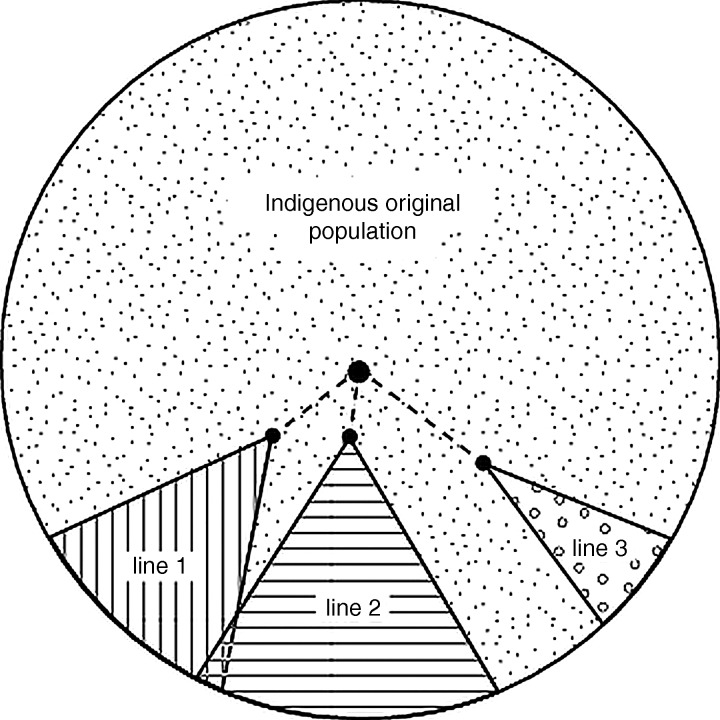
Diagram depicting the non-random distribution of arrhythmogenic right ventricular cardiomyopathy (ARVC) throughout the Boxer breed in the UK. The three family lines at risk of ARVC and deriving from males imported from the USA are shown, with the original disease-free section of the breed defined by exclusion

Finally, a third small family group (line 3) that is now effectively extinct was recognised. This too traced back to the Harpster study group (data not shown). Twenty-five dogs belonged in this line (12 males, 13 females). In that all lines traced back to a common source, it can be concluded that only one disease is involved. Importantly, no confirmed ARVC cases have been reported from outside these three lines over the 10-year span of the study such that the remaining indigenous population of Boxers in the show section of the breed in the UK can be deduced to be free of the gene or carry it at a very low frequency with a limited distribution (Fig 3).

### Inheritance

Inbreeding is standard procedure in show dog breeding. Accordingly, inbreeding particularly on one line 1 import (Fig 1, dog B) initially complicated elucidation of the mode of inheritance, this being further confounded by the low penetrance which made a skipping of generations virtually the norm. However, in later line 1 generations, outcrosses to the deduced normal population also produced the disease, establishing that ARVC has indeed a single gene autosomal dominant mode of inheritance as suggested previously (Meurs and others 1999). This pattern was also evident in line 2. Attempting to estimate the penetrance was problematical because of the difficulty in monitoring litters over many years. However, indications of very low penetrance were given by: (1) the observation that transmitting parents were seldom found to develop the disease (Figs 1 and 2), although checking for disease development in older animals is uncertain; (2) despite the 50 per cent frequency of affected animals expected with a dominant gene inheritance, few litters contained more than a single case manifesting clinical signs (mean Boxer litter size approximately 6.5) and (3) Holter confirmation of developing ARVC was prospectively sought with three aged, symptom-free ARVC-transmitting dogs (screened at 9, 10 and 13 years, showing evidence of ventricular arrhythmia with a ‘left bundle-branch block’ morphology and no other clinical abnormalities), thus confirming their pedigree genotyping as well as directly illustrating the low penetrance. The nine-year-old dog had a normal 24AECG initially, but this was abnormal when retested at 10 years of age. The penetrance in UK Boxers appears to be far lower than that reported by Meurs and colleagues (2010), who estimated it to be approximately 80 per cent in STRN heterozygotes and nearly 100 per cent in homozygotes. The discordance may reflect the different method of assessment: direct observation on parents of ARVC cases over many generations in our studies versus numbers of ARVC-affected dogs as a proportion of a total that expresses STRN in Meurs and others (2010) data.

### STRN disease association

**FIG 4:**
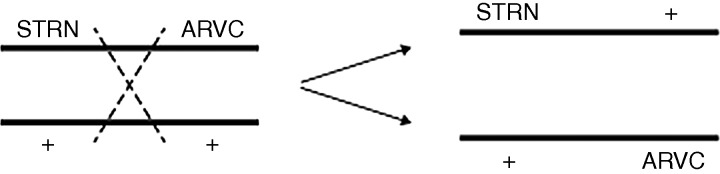
Diagram depicting recombination between the STRN and the arrhythmogenic right ventricular cardiomyopathy (ARVC) loci. Recombination will only be detectable when the transmitting parent is heterozygous at both STRN and ARVC loci, for example, STRN ARVC/++ (mutations in coupling), or STRN+/+ ARVC (mutations in repulsion). Recombination in STRN ARVC/++ parents will yield STRN+/+ ARVC recombinant progeny, and vice versa, but recombinants can only be detected when they are homozygous for an STRN allele, as this is the only way of being sure that the allele in question is located on the same chromosome as the ARVC mutation (see Fig 2). For this reason, STRN heterozygotes are not considered for detecting recombination

STRN genotyping of dogs in line 1 showed a clear association between the mutation and the ARVC phenotype (Table 1 and Fig 1) as described by Meurs and others (2010). Thus, all the affected dogs were either homozygous or heterozygous for the STRN mutation. However, by contrast, there was no suggestion of a STRN disease association in line 2 dogs where WT, as well as STRN homozygotes (HOM) and heterozygotes (HET), were found (Fig 2, heterozygotes not shown, Table 1). These data, in Boxers of close common origin, suggest that the STRN mutation itself is not the cause of the disease as proposed (Meurs and others 2010), but provides genetic evidence that a mutation in another gene lying close to it on the same chromosome is responsible. Meiotic recombination (Fig 4) can then account for the observed loose association between the STRN locus and the ARVC phenotype, with affected dogs being able to be WT, HOM or HET for the STRN mutation. Support for this is the indication of an inherited association between the WT allele of STRN and ARVC in a major section in line 2 (Fig 2) and the observed switching within line 2 from one association to the other. We have also confirmed the occurrence of STRN genotypes HOMs, HETs and WTs in affected and phenotypically normal dogs (even with an excess of HOMs in the Holter-tested group) (Table 1; Dukes-McEwan and others 2010), showing that the STRN mutation does not segregate with the disease status.

**TABLE 1:** STRN genotypes observed in Boxer Family line 1, line 2 and the normal disease-free section of the breed and comparison with randomly sampled Holter-tested cases and controls

	HOM	HET	WT	Total
Line 1 ARVC cases	10	13	0	23
Line 2 ARVC cases	6	8	10	24
Normal (by pedigree)	5	16	10	31
Holter-tested ARVC cases*	5	14	7	26
Normal (Holter tested)*	6	3	4	13

*Data from Dukes-McEwan and others (2010). Small sample size and different selection criteria in the two normal groups may account for the differing STRN mutation frequencies and the Hardy-Weinberg near-disequilibrium in the Holter-tested normal group (P(null)=0.057). However, there was not a statistically significant difference between the frequencies of the genotypes in the two normal groups (P=0.081). The main point is that the STRN mutation is common in both samples of normal dogs. There is a significant difference between the frequency of genotypes in lines 1 and 2 (P=0.002). However, there is no statistically significant difference in the frequency of genotypes between all ARVC cases and all normals (P=0.0597)

ARVC, arrhythmogenic right ventricular cardiomyopathy; HOM, homozygous for the STRN deletion mutation; HET, heterozygous for the STRN mutation; WT, wild type (normal)

### Harpster categories of ARVC and age of diagnosis

From Figs 1 and 2 and Table 1, it can be seen that all three estimated categories of ARVC severity can be found within each line and this makes it very unlikely that the severe Cat. 3 (DCM phenotype) is a genetically different condition. It can be best understood as a different expression of the same disease. However, although age of diagnosis and category were variable, there was a suggestion of an association between STRN homozygosity and more severe disease, but limited numbers of assessed dogs precluded further evaluation of this.

## Discussion

Most of the information presented here derived from a study on the incidence and distribution of ARVC in the show section of the Boxer breed in the UK, which was conducted primarily with a view to establishing a breeding control scheme for Boxer owners. Although there is no experimental component to this study, a number of important conclusions can be drawn concerning aspects of the disease.

### Origins

It was possible to trace the origins of the disease in each line by tracking pedigrees back through transmitting dogs to show that a small number of breeding animals imported from America were the source of the disease in the UK and that each of these originated from, or was closely related to, the group of American ARVC dogs originally studied by Harpster (1983). It is therefore highly improbable that different mutations could be responsible for the disease in the different lines and in the two countries. An important corollary of this finding is that the sections of the breed in the UK that do not have these imported dogs in their ancestry can be deduced to be free of the gene or are at very low risk. These dogs represent the indigenous population (Fig 3), which is mostly of Continental European origin, and this is consistent with anecdotal reports from veterinary conferences that Boxers of Continental breeding are largely free of the disease. The pedigree analysis is consistent with a single gene homogeneous disorder within the Boxer breed and this does not support the concept that genetic heterogeneity or phenocopies explain cases of inherited Boxer ARVC that do not carry the STRN mutation (Meurs and others 2010).

### Mode of inheritance and penetrance

On the basis of these findings, the breeding data show that affected dogs can inherit the disease from one parent only; a single gene autosomal dominant inheritance is therefore indicated, as has been suggested by Meurs and others (1999) and also seen with human ARVC (Iyer and Chin 2013). The penetrance cannot be reliably calculated but is indicated to be low by: (1) the low incidence of the disease in transmitting parents; (2) the clear shortage of affected progeny among the progeny of transmitting parents and (3) the detection by Holter of ARVC among tested older dogs deduced only from their breeding data to carry the gene. On this basis, the penetrance might appear to be closer to the 20–30 per cent estimate for ARVC dominant inheritance in humans (Sen-Chowdhry and others 2005) than the 80 per cent suggested by Meurs and others (2010).

### STRN and ARVC

The most important point indicated by the data presented here is that the STRN mutation is not responsible for the ARVC. Thus, although complete association between the mutation and phenotype was found in line 1 (Fig 1 and Table 1), no such concordance was found in line 2, which has the same recent ancestral origin. This line has WT as well as STRN HOMs and HETs among its numbers, and one section of the family also shows indications of an inherited WT STRN allele–ARVC association (Fig 2 and Table 1). Moreover, all three STRN genotypes were observed among both normal groups (the tested group and the exclusively defined-by-ancestry group, Table 1). Both of these normal groups have breeding records that indicate freedom or low risk of ARVC, the defined-by-ancestry group has served as an effective control in a GWAS study (R M Hamilton, personal communication), and finally both groups are indicated to be effectively ARVC free by the fact that selective breeding into this population, which carries the STRN mutation in high frequency, has virtually eliminated ARVC from the show section of the UK Boxer population. This conclusion is based on both breeder experience and an absence of ARVC case reports from those veterinary surgeons who previously reported cases. The genetic findings do, however, verify that the disease gene is located on CFA 17 and that it lies close to STRN such that meiotic recombination (Fig 4) has been able to generate, and will continue to generate, WT and STRN genotypes in affected and unaffected dogs. The pedigree data, not available in Meurs and others’ (2010) work, provide the genetic evidence for the simple recombination interpretation and make other complex speculative interpretations, such as genetic heterogeneity, phenocopies due to non-genetic causes, or two ARVC loci on the same chromosome, highly unlikely. It should also be noted that to enable recognition of meiotic recombination, the hypothesis requires that the STRN mutation is segregating in the normal Boxer population, British and American. This has been verified in the UK (Dukes-McEwan and others 2010) and is also indicated in the defined-by-ancestry disease-free section of the breed employed in the present study (Table 1).

While this STRN conclusion is at variance with that of Meurs and others (2010), it is not at variance with their findings. They noted that 4/61 affected dogs did not have the mutation and 9/38 normal controls did (attributed to low penetrance), and recently, Meurs and others (2014) have also reported that 7/43 genotyped Boxers diagnosed with ARVC were negative for the STRN mutation (16 per cent). In addition, they identified the STRN locus in the smaller of two statistically significant GWAS peaks 4 Mb apart on CFA 17. In view of the STRN–ARVC causal locus recombination indicated here, it is likely that the larger peak encompasses the gene for ARVC despite the perceived absence of defined genes in this region (Meurs and others 2010). The data of Meurs and others (2010) and the pedigree data presented here both indicate that further scans for this second CFA 17 locus should be instigated.

### The consequences

The finding that the STRN mutation is not responsible for ARVC means that ARVC-affected dogs without the mutation are not being recognised as having the inherited ARVC, and many normal dogs with the mutation are erroneously being defined as carrying the disease. A further outcome from the conclusions is that homozygosity for the STRN mutation no longer indicates homozygosity for the actual ARVC mutation. The fate of these ARVC homozygotes is now unclear. However, STRN remains a loose marker for the ARVC gene and still identifies the gross region. It should therefore be possible to find DNA markers that are closer to the ARVC gene and that could therefore serve better for identifying dogs that carry it. Such an exercise might also help the detection of the gene itself, in humans as well as Boxers.

### A possible STRN effect

Our previous studies have shown that the age of diagnosis in homozygotes for the STRN mutation may be earlier than those with other STRN genotypes, although the difference was not statistically significant (Dukes-McEwan and others 2010). The trend can be visualised more explicitly in the pedigree charts presented here (Figs 1 and 2), with STRN HOM–WT comparisons being possible in line 2. For some of these dogs, Harpster severity categories have also been determined and a further association between these and the age of diagnosis is suggested Indeed, Meurs described the frequency of ventricular ectopy (Meurs and others 2010) and the DCM phenotype (Meurs and others 2013) to be associated with the STRN mutation, and possibly with homozygosity, and this equally accords with the ARVC locus being close to STRN. Although an age-based progression of the disease possibly confuses severity assignments, examples of late-onset Cat. 1 cases can be found and these are consistent with the low genetic penetrance of the disease. Progression is recognised with the more severe Cat. 2 and 3 cases. As STRN plays a role in the cardiomyocyte, it may interact with the (unknown) ARVC mutation to trigger its development, as may exercise, myocarditis, stress or concurrent heart conditions. Furthermore, the STRN genotype may influence the severity or manifestation of other cardiac diseases.

### Limitations of this study

As with any mainly retrospective clinical study, there are potential limitations with this work. ARVC is not an easy disease to diagnose, and the guidelines for diagnosis in humans include consideration of the family history *alongside* imaging and ECG (Smith and others 2011). Only a proportion of our controls (free of ARVC disease or gene) were confirmed to be free of the disease by active phenotyping (echo, 24AECG), the others being deduced to be free by pedigree analysis (through having no evidence of disease in ancestors or offspring over many generations). However, although this might appear to be a source of inaccuracy, with a low penetrance, late-onset familial disease such as ARVC in Boxers, the pedigree-led approach is potentially more reliable than conventional clinical screening. An individual carrying the gene would be expected to show evidence of this somewhere in their ancestry or offspring if a sufficiently wide time span is considered, whereas they might appear to be completely normal when examined by ECG and 24AECG on only one or two occasions. Over-reliance on clinical screening, ignoring familial history, could easily have resulted in animals carrying the gene being erroneously included as controls in previous studies. The pedigree-led approach was further validated as it allowed the identification and targeted screening of some ‘discordant’ dogs, apparently healthy but known to transmit ARVC to their progeny, confirming the presence of Cat. 1 ARVC (asymptomatic ventricular arrhythmias with no other explanation) in these individuals.

Ventricular arrhythmias can be the result of other cardiac diseases and systemic conditions, which ideally should be excluded (e.g. by blood tests, abdominal and cardiac ultrasonography) before a diagnosis of ARVC is made. The ARVC cases were phenotyped as fully as possible, particularly the cases referred to the Liverpool and Glasgow Veterinary Hospitals. The BMC group of cases were seen by various different clinicians and cardiologists, but one author (PRW) reviewed all diagnoses for this group of ARVC cases, and where the diagnosis was in doubt that case was excluded. Only potential ARVC cases with a verifiable pedigree were included.

Independent of any of the aforementioned concerns about accurate phenotyping, the main conclusion of this study, namely that the STRN mutation is not the cause of ARVC in Boxers, is established from the observation of inherited WT ARVC lines of dogs within line 2, in addition to those carrying the STRN mutation. Clinically normal dogs carrying the STRN mutation have been found in all published Boxer ARVC studies using 24AECG testing (Dukes-McEwan and others 2010, Meurs and others 2014, Oxford and others 2014) as well as in the original work (Meurs and others 2010). Their presence was explained in these publications by incomplete penetrance, but here we suggest the presence of a separate, though linked, gene. With meiotic recombination between the STRN and a true ARVC locus on CFA 17, all STRN genotypes should be found in affected and normal Boxers, which is the case. Incomplete penetrance of the ARVC mutation is only an additional factor, and direct evidence for this is provided in this study.

## Conclusions

Pedigree studies indicate that Boxer ARVC in the UK has the same genetic basis as that in the USA. It appears to be caused by a single dominant gene mutation with incomplete penetrance located on CFA 17, but this is not the candidate gene STRN, the observed mutation of which can be present or absent within families of ARVC dogs, and is widely present in disease-free sections of the breed. Instead, STRN appears to serve only as a linked marker for an as yet undetected ARVC locus lying on the same chromosome and thus tends to be co-inherited with it, but the STRN–ARVC association is broken when there is meiotic recombination between the two genes. The severity categories and varying ages of diagnosis are indicated to be varying expressions of the one disease rather than attributable to different cardiomyopathies.
